# Ruptured appendiceal cystadenoma presenting as right inguinal hernia in a patient with left colon cancer: A case report and review of literature

**DOI:** 10.1186/1471-230X-6-32

**Published:** 2006-10-30

**Authors:** Yueh-Tsung Lee, Hurng-Sheng Wu, Min-Chang Hung, Shang-Tao Lin, Yome-Shine Hwang, Min-Ho Huang

**Affiliations:** 1 Department of Surgery, Division of general surgery, Chang-Bing Show Chwan Memorial Hospital, Lu-Gang, Taiwan; 2 Department of Surgery, Division of general surgery, Chang-Hua Show Chwan Memorial Hospital, Chang-Hua, Taiwan; 3 Department of Pathology, Chang-Hua Show Chwan Memorial Hospital, Chang-Hua, Taiwan

## Abstract

**Background:**

Mucoceles resulting from cystadenomas of the appendix are uncommon. Although rare, rupture of the mucoceles can occur with or without causing any abdominal complaint. There are several reports associating colonic malignancy with cystadenomas of the appendix. Herein, we report an unusual and interesting case of right inguinal hernia associated with left colon cancer.

**Case presentation:**

A case of ruptured mucocele resulting from cystadenoma of the appendix was presented as right inguinal hernia in a 70-year-old male. The patient underwent colonoscopy, x-ray, ultrasound and computed tomography. Localized pseudomyxoma peritonei associated with adenocarcinoma of the descending colon was diagnosed. The patient underwent segmental resection of the colon, appendectomy, debridement of pseudomyxoma and closure of the internal ring of right inguinal canal. He is free of symptoms in one year follow-up.

**Conclusion:**

Synchronous colon cancer may occur in patients with appendiceal mucoceles. In such patients, the colon should be investigated and colonoscopy can be performed meticulously in cases of ruptured mucoceles and localized pseudomyxoma peritonei. Surgical intervention is the current choice of management.

## Background

The incidence of mucocele ranges from 0.2–0.3% of all appendectomies and mucoceles resulting from cystadenomas of the appendix are very rare [1]. Although rare, rupture of the mucocele can occur with or without causing any abdominal complaint [2-4]. There are several reports associating colonic malignancy with mucocele of appendiceal cystadenoma [5-9]. We present a very unusual case of ruptured cystadenoma of the appendix with localized pseudomyxoma peritonei presenting as right inguinal hernia in a patient with left colon cancer.

## Case report

**Figure 1:**
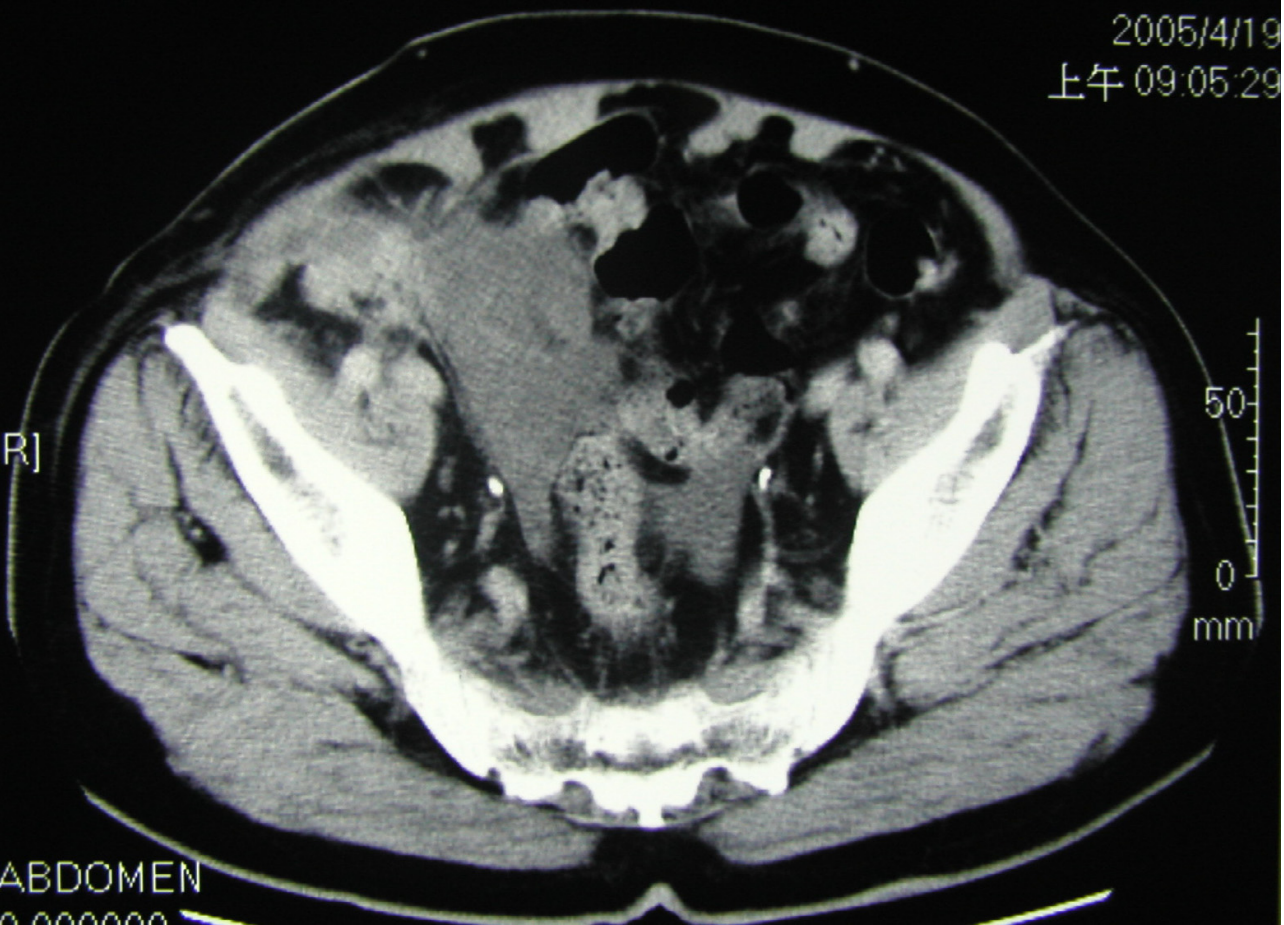
The CT scan showed the irregular hypodense lesion with fat stranding nearby the cecum without obvious enlarged lymph nodes.

**Figure 2:**
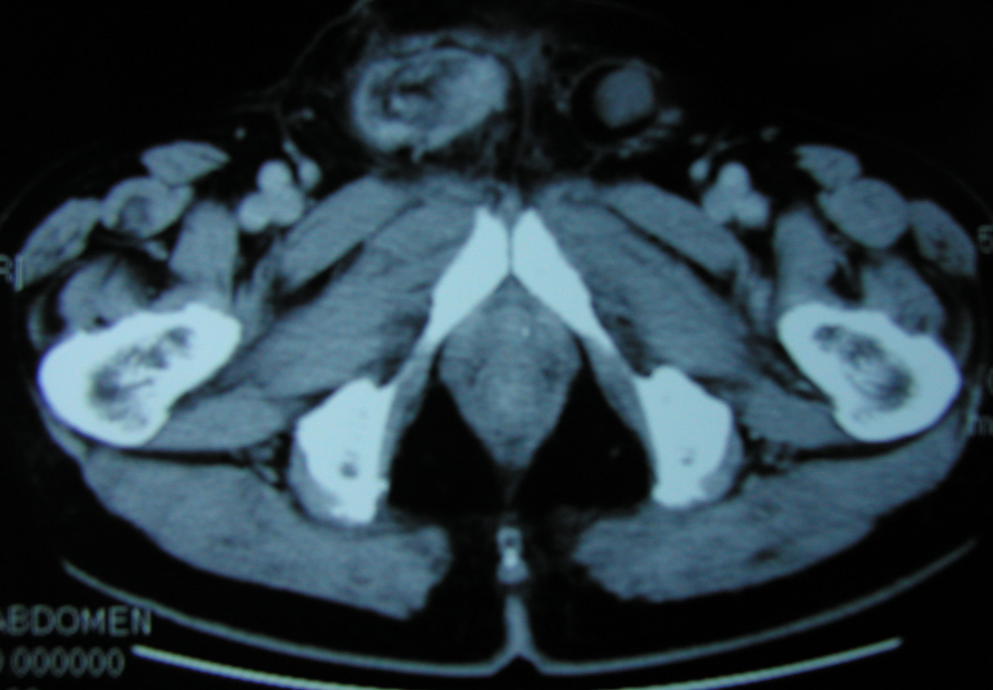
A heterogenous hypodense lesion with adjacent fat stranding in the right inguinal canal was noted on the CT scan.

**Figure 3:**
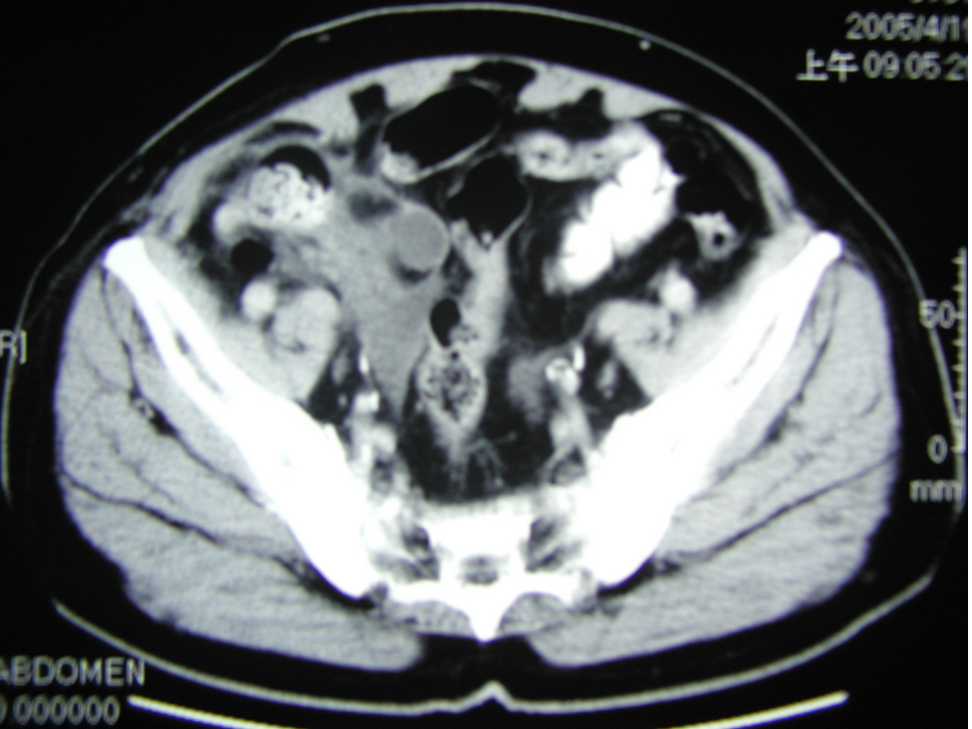
No oral contrast media leaking from the cecum into the peritoneal cavity was noted on the CT scan.

**Figure 4:**
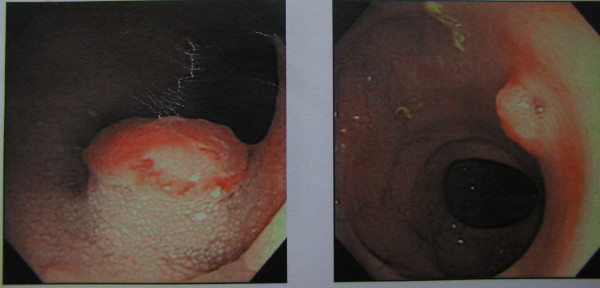
A sessile polyp, 2 cm in diameter, in the descending colon was discovered by colonoscopy.

**Figure 5:**
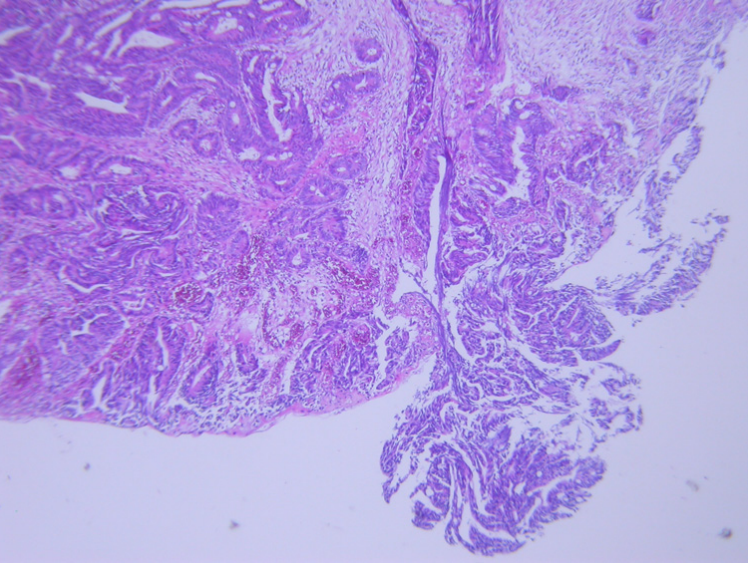
Microscopically, the tumor cells were large, hyperchromatic and pleomorphic with irregular glandular formation suggestive of moderately differentiated adenocarcinoma and invaded the muscle layer of colon.

**Figure 6:**
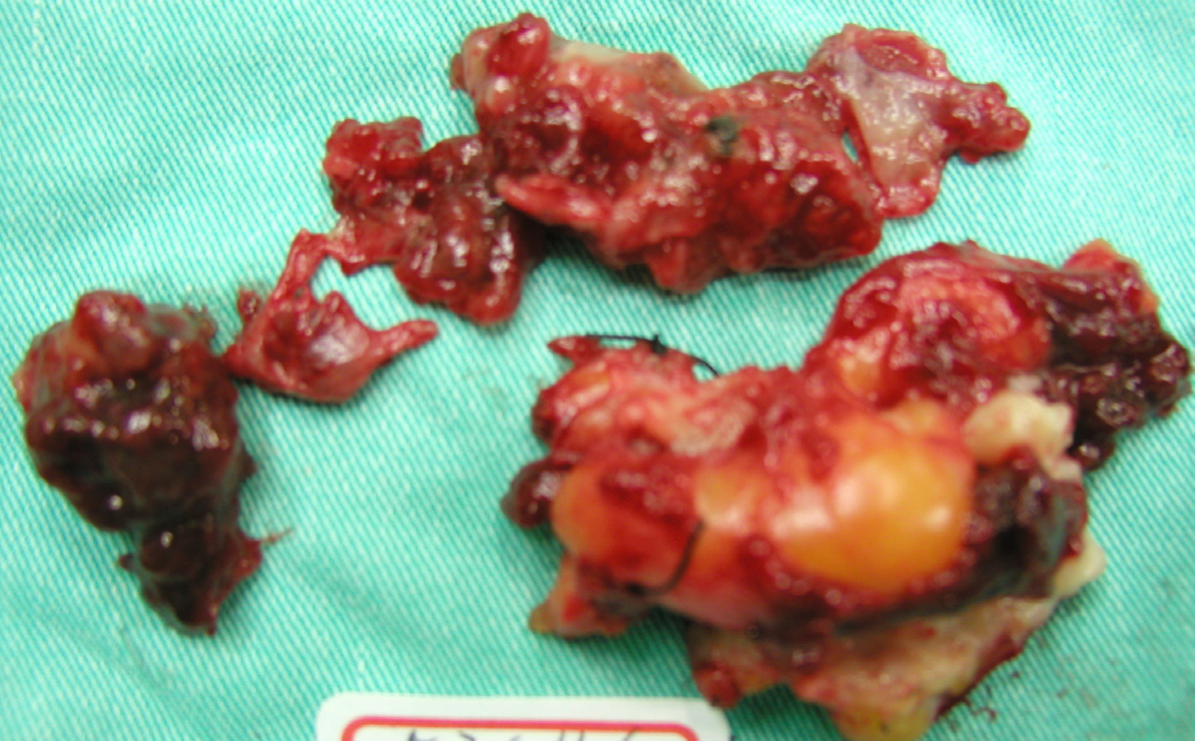
At laparotomy, the appendix was excised and the pieces were removed.

**Figure 7:**
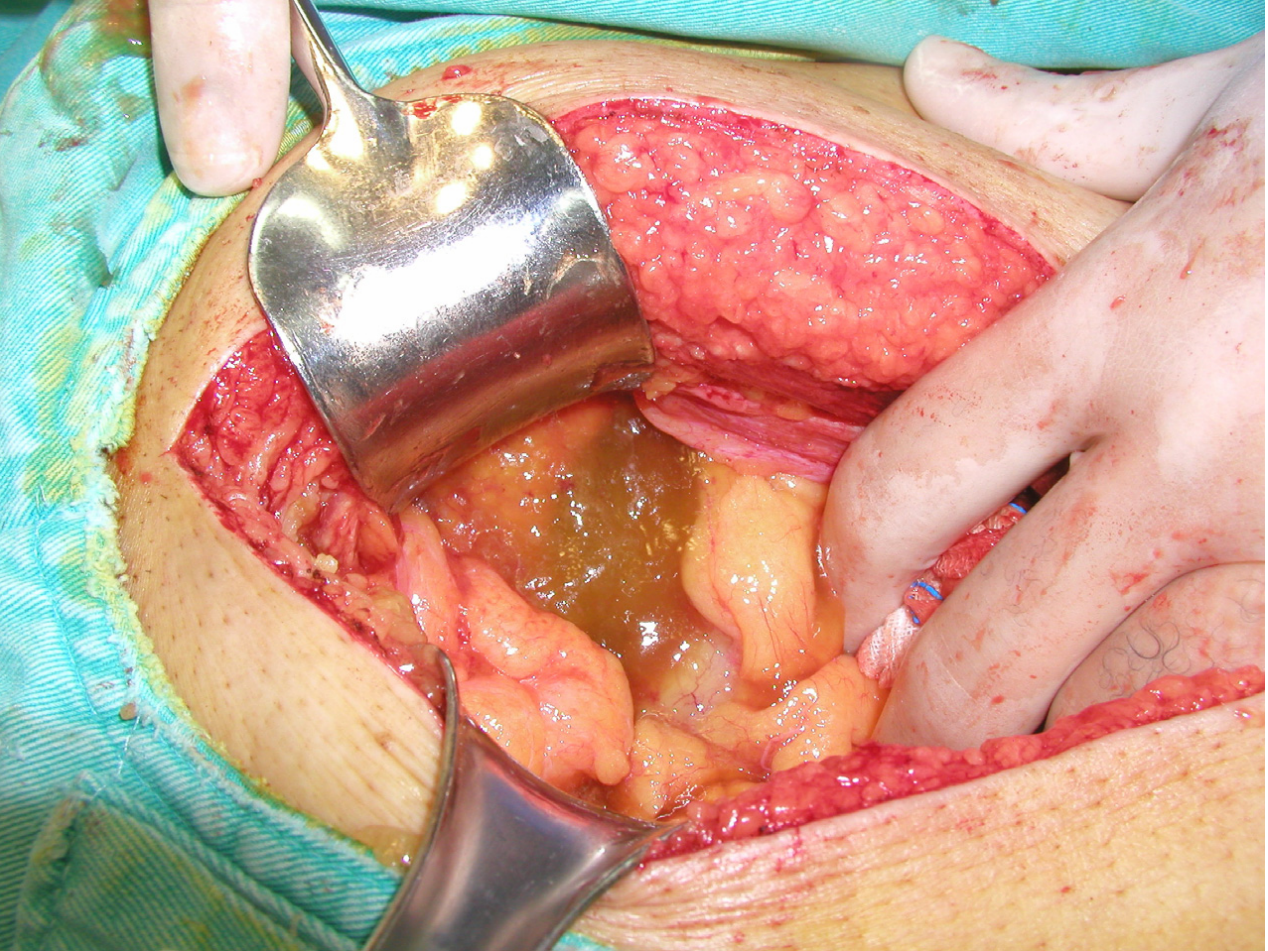
The lower peritoneal cavity and the right inguinal canal were filled with yellowish gelatinous fluid.

**Figure 8:**
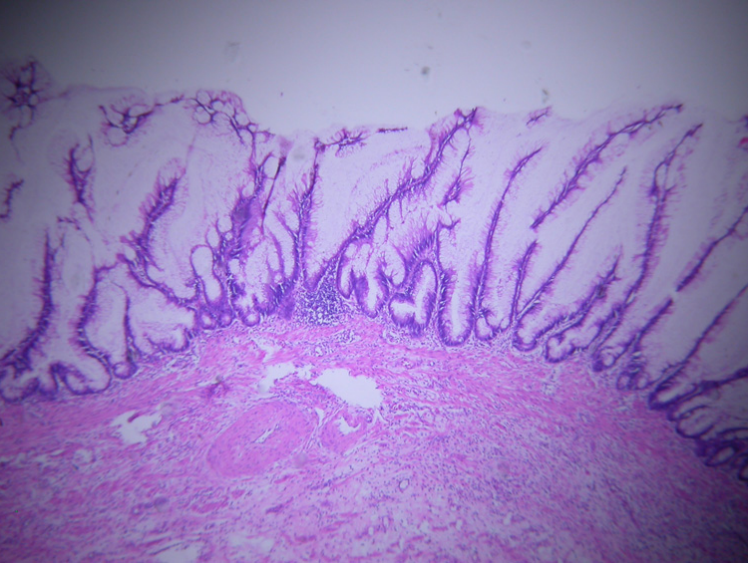
Microscopically, the mucosal glands of the appendix demonstrated dilatation and containing mucin suggestive of mucinous cystadenoma. The periappendix tissue showed mucinous tissue mixed with granulation tissue without tumor in it and consistent with pseudomyxoma peritonei.

A 70 year-old male presented with a right inguinal mass and dragging sensation over it for 2 months before admission to the hospital. He was on medication for hypertension and moderate aortic regurgitation for years with good control. We palpated a non-tender, irreducible and doughy mass over the right inguinal region. The laboratory data was within the normal limits including the tumor marker(CEA). Abdominal ultrasound showed an irregular cystic hypoechoic lesion over the right lower quadrant. CT scan revealed an irregular hypodense lesion with fat stranding near the cecum without obviously enlarged lymph nodes(Figure 1). The right inguinal canal was occupied by a heterogenous lesion with adjacent fat stranding suggestive of appendiceal mucocele with rupture(Figure 2). We noted that no leakage of oral intake of iodinated, water-soluble contrast medium from the cecum or appendix into the peritoneal cavity(Figure 3). We postulated no persistent leakage of mucin from the appendix. A gentle colonoscopic examination was performed. It revealed a 2 cm in diameter sessile polyp in the descending colon(Figure 4). Polypectomy was performed and the pathology revealed moderately differentiated adenocarcinoma with muscle layer invasion(Figure 5). At laparotomy, a segmental resection of the descending colon with excision of the mesenteric lymph nodes, appendectomy(Figure 6), removal of yellowish mucoid jelly(Figure 7) and closure of the internal ring of the right inguinal canal were performed. Pathology revealed cystadenoma of the appendix(Figure 8) and moderately differentiated adenocarcinoma of the descending colon, without regional lymph node metastases. The hospital course was uneventful. The patient is free of symptoms at one year follow-up.

## Discussion

Appendiceal mucocele causes the mucinous distention of the appendiceal lumen. The pathologic entity includes retention cyst, mucosal hyperplasia, cystadenoma and cystadenocarcinoma [10]. Symptomatic lesions are associated with malignant diseases more common than asymptomatic ones [11]. There are reports of other tumors associated with appendiceal mucoceles, including gastrointestinal tract, ovary, breast and kidney tumors, which might occur in up to one-third of the patients [12]. Pitiakoudis et al. have reported synchronous colonic cancer associated with appendiceal cystadenocarcinoma [13]. An increased incidence of colonic cancer in patients with appendiceal mucocele has been reported [14]. We reviewed other ten patients with appendiceal mucoceles undergoing appendectomies in our hospital during the past 13 years. Three of them had presented ruptured lesions. We recalled some of them for the colonoscopic examination at outpatient department. For the other patients who refused further colonoscopy, we investigated the history of colonic surgery, colonoscopic examination or bowel habit change during the past period. At presentation, we had no evidence of occurrence of concomitant colonic cancer in the patients. The concomitant pathologies are often clinically silent [1]. The present patient was also asymptomatic before the rupture of the mucocele and its presentation as right inguinal hernia. There are also reports associating ruptured appendiceal mucoceles with or without colonic cancer [2,3,14]. However, none of them presented as inguinal hernia. To the best of our knowledge, no such case has ever been reported. The more advanced lesions are associated with higher incidence of concomitant lesions [15]. Synchronous colonic lesions should also be looked for and these are more common in advanced lesions [16-18]. Therefore, colonoscopy is recommended as mandatory before surgery when possible [19]. However, colonoscopy can sometimes cause severe and fatal complications [20-22] and there are techniques recommended to perform the procedure meticulously [23,24]. We did not find oral intake of contrast medium leaking from the cecum on the CT scans. Therefore, we performed the procedure and found the distal colonic malignancy. The patient underwent surgery according the recommendation of others [12,25-27].

## Conclusion

Synchronous colon cancer may occur in patients with appendiceal mucoceles. In such patients, the colon should be investigated. In patients with ruptured mucoceles and localized pseudomyxoma peritonei, the colonoscopy can be performed meticulously. Surgery is the recommended method of treatment.

## Competing interests

The author(s) declare that they have no competing interests.

## Authors' contributions

YT Lee was in charge of the patient and did the surgery mainly. HS Wu and MH Huang were the consultants of the surgical plan. MC Hung and YS Hwang assisted the surgery. ST Lin was the reporter of pathology. All authors read and approved the final manuscript.

## Pre-publication history

The pre-publication history for this paper can be accessed here:


